# Mitigation of Radiation-Induced Lung Pneumonitis and Fibrosis Using Metformin and Melatonin: A Histopathological Study

**DOI:** 10.3390/medicina55080417

**Published:** 2019-07-30

**Authors:** Bagher Farhood, Akbar Aliasgharzadeh, Peyman Amini, Abolhasan Rezaeyan, Alireza Tavassoli, Elahe Motevaseli, Dheyauldeen Shabeeb, Ahmed Eleojo Musa, Masoud Najafi

**Affiliations:** 1 Departments of Medical Physics and Radiology, Faculty of Paramedical Sciences, Kashan University of Medical Sciences, Kashan 8715988141, Iran; 2 Department of Radiology, Faculty of Paramedical, Tehran University of Medical Sciences, Tehran 1416753955, Iran; 3 Department of Medical Physics, School of Medicine, Iran University of Medical Sciences, Tehran 1449614535, Iran; 4 Department of Pathology, Fasa University of Medical Sciences, Fasa 8668874616, Iran; 5 Department of Molecular Medicine, School of Advanced Technologies in Medicine, Tehran University of Medical Sciences, Tehran 1416753955, Iran; 6 Department of Physiology, College of Medicine, University of Misan, Misan 62010, Iraq; 7 Department of Medical Physics and Biomedical Engineering, Faculty of Medicine, Tehran University of Medical Sciences (International Campus), Tehran 1416753955, Iran; 8 Radiology and Nuclear Medicine Department, School of Paramedical Sciences, Kermanshah University of Medical Sciences, Kermanshah 6715847141, Iran

**Keywords:** radiation, mitigation, lung, melatonin, metformin, fibrosis, pneumonitis

## Abstract

*Background and objectives*: Pneumonitis and fibrosis are the most common consequences of lung exposure to a high dose of ionizing radiation during an accidental radiological or nuclear event, and may lead to death, after some months to years. So far, some anti-inflammatory and antioxidant agents have been used for mitigation of lung injury. In the present study, we aimed to detect possible mitigatory effects of melatonin and metformin on radiation-induced pneumonitis and lung fibrosis. *Materials and methods*: 40 male mice were divided into 4 groups (10 mice in each). For control group, mice did not receive radiation or drugs. In group 2, mice were irradiated to chest area with 18 Gy gamma rays. In groups 3 and 4, mice were first irradiated similar to group 2. After 24 h, treatment with melatonin as well as metformin began. Mice were sacrificed after 100 days for determination of mitigation of lung pneumonitis and fibrosis by melatonin or metformin. *Results*: Results showed that both melatonin and metformin are able to mitigate pneumonitis and fibrosis markers such as infiltration of inflammatory cells, edema, vascular and alveolar thickening, as well as collagen deposition. *Conclusion*: Melatonin and metformin may have some interesting properties for mitigation of radiation pneumonitis and fibrosis after an accidental radiation event.

## 1. Introduction

Pneumonitis and fibrosis are the most common consequences of lung exposure to high dose of ionizing radiation. Pneumonitis refers to acute inflammation and edema of lung tissues which lead to severe problems in breath and may lead to death. Pneumonitis occurs following the release of a large amount of inflammatory cytokines from macrophages and lymphocytes [1,2]. Irradiation of lung tissue leads to DNA damage and cell death which trigger release of chemokines and cytokines, resulting to accumulation of macrophages and lymphocytes in alveolar. If exposed to a high dose of radiation, infiltration of inflammatory cells will be severe, resulting to massive cell death and oxidative injury. Macrophages, lymphocytes and neutrophils release a large number of cytokines such as IL-1, IL-2, IL-4, IL-6, IL-8, IL-10, IL-13, IL-18, IL-33, TGF-β, TNF-α, IFN-γ, etc. These cytokines stimulate several signaling pathways which lead to the release of nitric oxide (NO), reactive oxygen species (ROS), prostaglandins (PGs) and some other mediators which lead to more oxidative injury and appearance of inflammatory reactions [3,4]. In addition to pneumonitis, chronic infiltration of inflammatory cells and continuous production of free radicals and some cytokines such as IL-4, IL-13, TGF-β, and IFN-γ, lead to upregulation of pro-oxidant and pro-fibrotic genes, resulting in accumulation of collagen and incidence of lung fibrosis. Fibrosis is an irreversible phenomenon which is associated with a high risk of death [5,6].

Although hematopoietic and gastrointestinal systems are the most radiosensitive organs, many studies in recent years suggested the importance of other organs such as lung, heart, brain, liver, skin and kidneys [7]. Developments in stem cell therapy have shown improvements in survival of people that were exposed to radiation up to 10 Gy [8,9]. However, exposure to a radiation dose of higher than 7–8 Gy may lead to kidney and liver failures, as well as incidence of lung pneumonitis, which poses a threat to the lives of irradiated people [10]. These issues are of utmost concern in radiotherapy, and also nuclear or radiological disasters. In recent years, several experimental studies have been conducted to mitigate lung injury through modulation of factors involved in radiation-induced pneumonitis and fibrosis [11,12,13].

Metformin is one of the most common anti-diabetic drugs that has shown potential antioxidant and radioprotective properties [14]. Melatonin is a natural antioxidant that regulates the circadian cycle in the body. It has shown ability to modify radiation response through several mechanisms such as antioxidant and anti-inflammatory effects [15]. The clinical efficacy of melatonin has also been investigated [16]. In the current study, we aimed to detect possible mitigatory effect of melatonin and metformin on radiation-induced pneumonitis and fibrosis markers when administered after irradiation of lung tissues. We hypothesized that potential antioxidant, anti-inflammation and DNA repair stimulating effects of these agents which have been confirmed in previous studies can help alleviate both pneumonitis and fibrosis. Thus, it may be proposed for lung cancer radiotherapy patients, as well as people who have been exposed to an accidental radiation event.

## 2. Materials and Methods

### 2.1. Chemical Agents

Melatonin (98%) was purchased from Sigma Aldrich Company. Melatonin powder was dissolved in 70% ethanol and then diluted using distilled water. The final concentration of ethanol was 15% while that of melatonin was 3 mg per milliliter. Metformin (98%) was provided by Tehran Chemie Pharmaceutical Company, Tehran, Iran. It was dissolved in distilled water at a similar concentration with melatonin solution. For each drug treatment, in each day, fresh solutions of both melatonin and metformin were provided. Treatments with both melatonin and metformin were performed as 100 mg/kg body weight. For administration of melatonin or metformin, mice received 1 mL melatonin or metformin solution orally each day, for 5 days per week.

### 2.2. Irradiation

Mice (30 g body weight) were irradiated using a cobalt-60 (^60^Co) gamma rays source at Imam Khomeini hospital, Tehran University of Medical Sciences, Tehran, Iran. Before irradiation, mice were anesthetized using ketamine and xylazine at 20 and 5 mg/kg body weight, respectively. The chest areas of mice were irradiated with gamma rays at a source to skin distance (SSD) of 60 cm and a dose rate of 94 cGy per minute. The total dose of gamma rays received by lung tissues was 18 Gy.

### 2.3. Experimental Design

40 male mice weighing 30 g were kept in standard conditions as 12 h light/12 h dark, 55% humidity, and temperature of 25 °C. Mice were divided into 4 groups (10 mice in each group). Group 1: Mice received only anesthesia drugs (ketamine and xylazine at 20 and 5 mg/kg body weight, respectively); Group 2: mice received a single dose of 18 Gy gamma rays to chest area; Group 3: similar to group 2, mice received 18 Gy gamma rays to chest area, and after 24 h, metformin treatment began for 2 weeks; Group 4: similar to groups 2 and 3, mice received 18 Gy gamma rays to chest area, and after 24 h, melatonin treatment began for 2 weeks. After 100 days, all mice were sacrificed, followed by the removal of their lung tissues.

### 2.4. Histopathological Evaluation

Lung tissues were fixed in 10% normal buffer formalin and then embedded into paraffin blocks. 4-micron thickness of samples were provided using microtome, and then stained with hematoxicilin and eosin (H&E), and masons thrichrome (MTC). Slides were evaluated by a Histopathologist for pneumonitis and fibrosis markers. For all evaluations, a light microscope (Olympus BX53, Tokyo, Japan) with ×100 magnification was used. The microscope’s field of view was 22 for ocular lens and 5.512 for objective lens. Thereafter, results were reported and scored according to our previous study [17].

### 2.5. Statistical Analysis

All statistical analyses were done using SPSS software version 22 (IBM, Chicago, IL, USA). Results for each group were calculated. Afterwards, their means and standard deviations were obtained. The differences between means were calculated and considered significant for *p* value < 0.05.

### 2.6. Ethical Approval

This study was under ethical code IR.KAUMS.NUHEPM.REC.1397.006, approved by Kashan University of Medical Sciences. Approval Date was 7 May 2018.

## 3. Results

**Figure 1:**
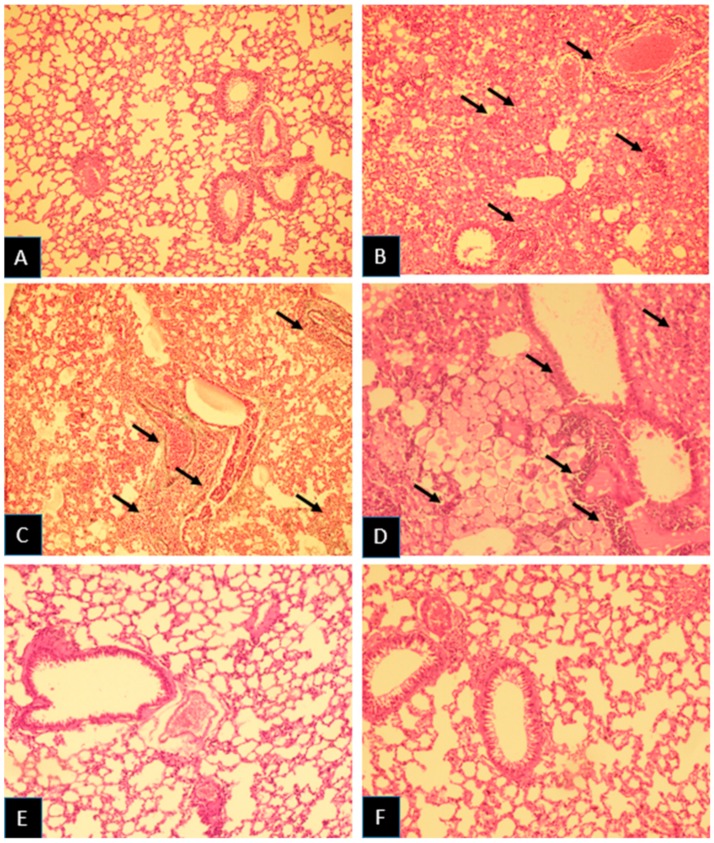
Histopathological investigation of the radio-mitigation effects of melatonin and metformin, and radiation damage after 100 days. (**A**) Control: Normal appearances of alveolar space, bronchioles, and vascular bed were observed. (**B**) Radiation: Severe interstitial inflammation and pulmonary edema were observed. (**C**,**D**) Radiation: Severe inflammation of bronchial wall was observed with destruction of bronchus. (**E**) Melatonin: Mild inflammation was observed. (**F**) Metformin: Mild inflammation was observed. The arrows indicate an accumulation of lymphocytes, macrophages, and neutrophils in lung tissues (H and E, ×100).

**Figure 2:**
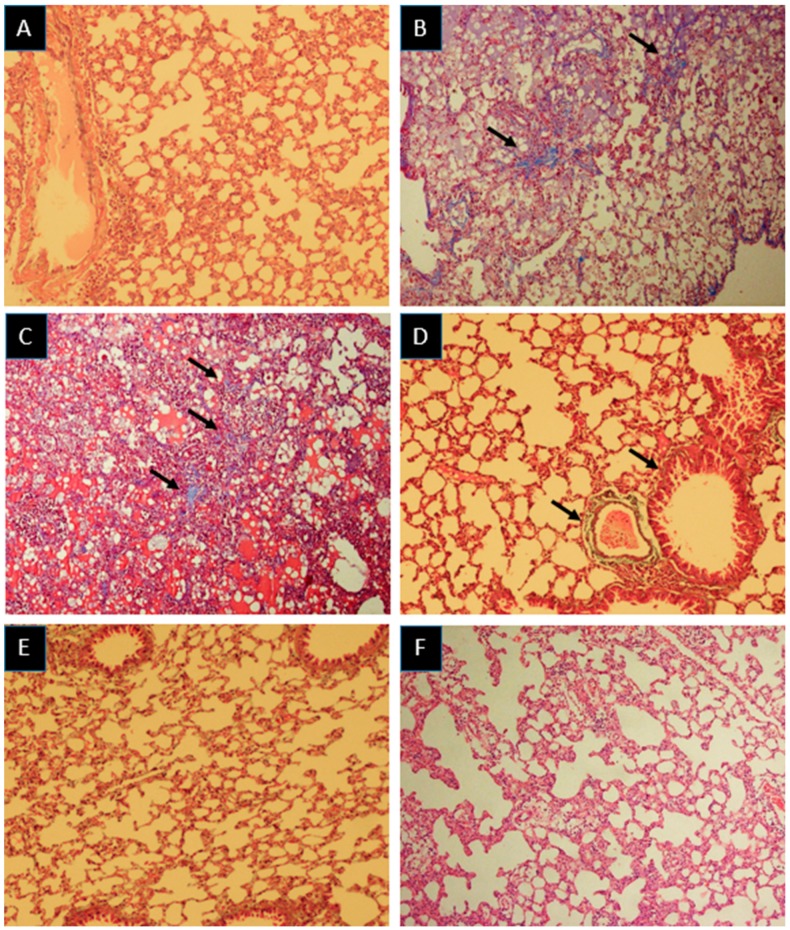
Histopathological investigation of the radio-mitigation effects of melatonin and metformin, and radiation damage after 100 days in the lung tissues. (**A**) Control: Normal appearances of alveolar space, bronchioles and vascular bed were observed. (**B**,**C**) Radiation: Moderate collagen deposition was observed. (**D**) Melatonin: Collagen deposition was mild around the vascular bed and alveolar spaces. (**E**,**F**) Metformin: Mild collagen deposition was observed. Collagen deposition is shown in light blue. The arrows indicate the collagen deposition in the lung tissues (Masson’s trichrome staining: Magnification ×100).

Histopathological results showed that irradiation of mice with 18 Gy gamma rays led to remarkable changes in the lung tissues. Results also showed that irradiation with 18 Gy gamma rays caused severe congestion, inflammation, infiltration of macrophages, lymphocytes and neutrophils, as well as edema. Treatment with melatonin post-irradiation led to alleviation of these damages. Also, irradiation led to mild to moderate vascular and alveolar damages, which were significantly reduced after melatonin treatment. Lung irradiation also led to moderate fibrosis, which was reversed completely following treatment with melatonin. Similar to melatonin, metformin showed ability to mitigate congestion and infiltration of macrophages, while it could not reduce inflammation or infiltration of lymphocytes and neutrophils. Furthermore, metformin treatment post irradiation led to alleviation of vascular and alveolar thickness, as well as edema. Similar to melatonin, treatment with metformin reversed fibrosis completely (Table 1, Figure 1 and Figure 2).

## 4. Discussion

In previous studies, we showed that treatment with metformin or melatonin before irradiation can reduce late effects of exposure to ionizing radiation in the lung. We also showed that melatonin and metformin reduce the release of IL-4 and its downstream pro-oxidant enzymes such as dual oxidase 1 and 2 (Duox1 and Duox2) [18,19]. In contrast to our previous studies, for the first time, in the present study we investigated the mitigatory effects of melatonin and metformin on pneumonitis and fibrosis of the lung following administration of these drugs after irradiation. Lung injury including pneumonitis and fibrosis, pose a threat to the lives of people who have been exposed to an accidental radiological event or nuclear disasters. They can occur following non-uniform whole body exposure of the lung to a high dose of radiation, while the lower parts of the body such as gastrointestinal system or bone marrow received sub-lethal doses of ionizing radiation [10]. In these conditions, an appropriate radiation mitigator may be useful to save the lives of exposed people. Furthermore, since lung pneumonitis and fibrosis appear some months to years after the end of treatment, a radiation mitigator can also be useful for patients who had undergone radiotherapy, too [20,21].

In the present study, we hypothesized that the anti-inflammatory and antioxidant properties of melatonin can alleviate lung injury after exposure to an acute radiation dose. So far, several anti-inflammatory and antioxidant agents have been used for mitigation of pneumonitis and lung fibrosis. Inhibition of inflammatory mediators such as COX-2 or NF-kB have been proposed for mitigation of lung injury [22]. Treatment with celecoxib, which inhibits COX-2 selectively, was able to mitigate death because of pneumonitis in mice [23]. Melatonin, via inhibition of toll like receptors (TLRs), COX-2 and NF-kB, has abilities to reduce the release of inflammatory cytokines and chemokines. These are associated with preventing recruitment of infiltration of macrophages, neutrophils and lymphocytes, which prevent continuous oxidative damage following respiratory burst by inflammatory cells [24,25]. Recently, it has been shown that suppression of miR-30e/NLRP3 in macrophages by melatonin attenuates lung injury [26]. Similar effect has been observed for melatonin in other tissues including the small intestine and tongue [27,28]. This shows that melatonin prevents upregulation of inflammasome/NLRP3 and pro-inflammatory cytokines such as IL-1, via protection of the mitochondria. In addition to inflammatory mediators and cytokines, it has been suggested that overexpression of ROS generating enzymes plays a key role in the development of pneumonitis and fibrosis. In the lung, it has been shown that some pro-oxidant enzymes such as NADPH oxidase-1 (NOX1), NOX2, NOX4, as well as Duox1 and Duox2 are involved in oxidative stress and development of fibrosis. Melatonin can attenuate the regulation of NOX2, NOX4, Duox1 and Duox2 [24,29,30]. Attenuation of these enzymes’ activities by melatonin is associated with reduced level of oxidative stress, infiltration of macrophages and lymphocytes, as well as suppression of fibrosis [24,29]. As these enzymes play a key role in vascular injury and infiltration of inflammatory cells within the alveolar, it is possible that melatonin, via suppression of these enzymes, reduces vascular and alveolar injuries [31,32]. Results of this study confirmed mitigation of lung injury by melatonin via alleviation of vascular and alveolar damage, edema, as well as reducing infiltration of inflammatory cells.

Metformin was another drug that we used to mitigate radiation-induced pneumonitis and lung fibrosis. Results showed that metformin can attenuate pneumonitis markers including infiltration of inflammatory cells, as well as damages to alveolar and vascular. Furthermore, similar to melatonin it could reverse moderate fibrosis completely. Metformin as a potent mitochondrial electron transformer chain (ETC), suppresses the endogenous production of free radicals by the mitochondria [33,34]. On the other hand, targeting the mitochondria has been proposed as a strategy for mitigation of radiation-induced lung injury [35]. Inhibition of the mitochondria can ameliorate cell death and reduce ROS generation as well as upregulation of pro-fibrosis cytokines such as TGF-β [35,36]. Post-irradiation treatment with metformin may attenuate the continuous production of free radicals from the mitochondria. In addition, metformin can attenuate chronic production of ROS and alleviates fibrosis, through modulation of pro-oxidant genes such as NOX4 [37]. Metformin has shown ability to attenuate radiation-induced toxicity via neutralization of free radicals, stimulation of antioxidant defense enzymes as well as stimulation of DNA repair enzymes [38,39]. It has also shown ability to trigger DNA damage responses (DDR), leading to protection of cells against toxic agents including ionizing radiation [40].

Results of this study confirmed that both melatonin and metformin can be used as potential radiation mitigators for alleviation of pneumonitis and fibrosis following exposure of the lung to an acute high dose of radiation. In the future, it is necessary to examine the possible mechanisms for mitigation of pneumonitis and fibrosis using these agents. Furthermore, using a combination of these drug with others can be more effective for this aim. For example, it has been revealed that abnormal upregulation of renin-angiotensin system plays a key role in both pneumonitis and fibrosis [41]. The combination of a renin-angiotensin system with an antioxidant may be more useful for mitigation of lung injury [42]. Moreover, administering these drugs at different times after irradiation can improve inhibition of inflammatory and fibrosis responses. In the present study, we administered melatonin and metformin for two weeks after irradiation, based on a previous study. However, further studies have shown challenging results.

## 5. Conclusions

This study has been conducted to determine the possible mitigatory effects of melatonin and metformin on radiation-induced lung injury. Treatment with both melatonin and metformin showed abilities to alleviate pneumonitis and fibrosis markers. In this study, we treated mice for 2 weeks after irradiation. However, it seems that using different times and drug combinations may be more useful for achieving best results. Our findings showed that melatonin and metformin may have some interesting properties for mitigation of radiation pneumonitis and fibrosis after an accidental radiation event.

## Figures and Tables

**Table 1:** Results of histopathological evaluation of lung injury following irradiation and post-irradiation treatment with melatonin or metformin.

	Control	RAD	RAD + MLT	RAD + MET
Erythrocyte	0.00 ± 00	3.33 ± 0.47 ^a^	0.00 ± 00 ^b^	0.00 ± 00 ^b^
Congestion	0.00 ± 00	4.00 ± 0.00 ^a^	1.60 ± 0.43 ^b^	1.50 ± 1.11 ^b^
Inflammation	0.00 ± 0.00	3.33 ± 0.57 ^a^	2.00 ± 1.14	2.66 ± 0.57
Macrophages infiltration	0.25 ± 0.43	3.00 ± 00 ^a^	1.50 ± 0.57 ^b^	1.50 ± 0.57 ^b^
Lymphocyte infiltration	0.00 ± 00	2.75 ± 0.96 ^a^	1.50 ± 1.00	1.66 ± 0.57
Neutrophil infiltration	0.00 ± 00	3.00 ± 0.81 ^a^	1.50 ± 0.57 ^b^	1.66 ± 0.57
Vascular wall thickness	0.00 ± 00	1.33 ± 0.57 ^a^	1.00 ± 00 ^b^	0.00 ± 00 ^b^
Vascular damage	0.00 ± 00	2.50 ± 0.50 ^a^	0.60 ± 0.54 ^b^	1.50 ± 0.57
Alveolar thickness	0.00 ± 00	1.00 ± 00 ^a^	0.25 ± 0.50 ^b^	0.25 ± 0.50 ^b^
Edema	0.00 ± 00	3.00 ± 00 ^a^	1.00 ± 1.41 ^b^	0.50 ± 0.57 ^b^
Fibrosis	0.00 ± 00	2.00 ± 00 ^a^	0.00 ± 00 ^b^	0.00 ± 00 ^b^

RAD—radiation; MLT—melatonin; MET—metformin. ^a^ Significant compared to control; ^b^ significant compared to radiation group. Results of histopathological evaluations were scored from 0 (normal) to 4 (very severe injury). Furthermore, results for each group were reported as mean ± standard deviation. Significant differences between groups were calculated using Mann—Whitney non-parametric test (*p* < 0.05).
